# Factors associated with advance directive completion among older adults with and without cognitive impairment: a nationwide cross-sectional study

**DOI:** 10.3389/fpubh.2026.1957140

**Published:** 2026-10-23

**Authors:** Seunghye Choi, Hana Ko

**Affiliations:** College of Nursing, Gachon University, Incheon, Republic of Korea

**Keywords:** advance care planning, advance directives, aged, cognitive dysfunction, dying well

## Abstract

**Objective:**

This study aimed to identify and compare the factors associated with advance directive (AD) completion between older adults with and without cognitive impairments, using a nationwide cross-sectional dataset.

**Methods:**

This study analyzed cross-sectional nationwide data obtained from the 2023 National Survey of Older Koreans, which is conducted every 3 years by the Korean Ministry of Health and Welfare. The study measured cognitive function in a total of 9,951 participants, using the Korean version of the Mini-Mental State Examination for Dementia Screening (K-MMSE-DS). The participants were classified into cognitive impairment (*n* = 2,448) and normal cognition (*n* = 7,503) groups, and the differences in AD completion based on demographic characteristics, health-related characteristics, and attitudes toward death of the participants in each group were analyzed using the chi-square test. A multivariable logistic regression model was used to identify the factors associated with AD completion.

**Results:**

Multivariable logistic regression analysis showed that religious affiliation, poorer nutritional status, higher exercise frequency, participation in death preparation education, and higher awareness of dying well were commonly associated with AD completion in both the cognitive impairment and normal cognition groups. Group-specific associations were also identified: in the cognitive impairment group, AD completion was additionally associated with younger age, female sex, higher education, increased prescription medication use, higher independence in activities of daily living, and higher depressive symptom scores, whereas in the normal cognition group, it was additionally associated with a higher number of chronic diseases and history of falls within the past year.

**Conclusion:**

Different patterns factors associated with AD completion were observed between older adults with and without cognitive impairment, suggesting that strategies to promote AD completion may need to be tailored according to cognitive status.

## 1 Introduction

With advancements in medical science and rapid population aging, South Korea has transitioned into a super-aged society, intensifying social demands for a “dignified death” and high-quality end-of-life care (1). As of 2025, approximately 10.5 million individuals in South Korea are aged 65 years or older, with an estimated 955,584 (9.09%) living with dementia (2). Furthermore, mild cognitive impairment affects approximately 28.09% of older adults, representing a substantial population at an elevated risk of developing dementia (2). Given the aging population and increasing prevalence of dementia, a growing proportion of older adults are at risk of losing their decision-making capacity (3). In this context, Advance Care Planning (ACP)—a process through which individuals, together with their families and healthcare providers, discuss and document preferences for future care, including for situations in which decision-making capacity may be lost—has become increasingly important (4). As one of the key components of ACP, Advance Directives (ADs) allow individuals to specify their preferences regarding future medical treatment, and have emerged as an important mechanism for protecting patient autonomy and improving the quality of end-of-life care (5). In South Korea, AD completion has been legally institutionalized under the Act on Hospice-Palliative Care and Decisions on Life-Sustaining Treatment for Patients at the End of Life, which took effect in 2018 and enables individuals to document their preferences regarding life-sustaining treatment in advance (6).

Dementia is characterized by a progressive, longitudinal disease trajectory in which cognitive function gradually deteriorates; therefore, ACP should be approached as an ongoing, dynamic process (7). Ideally, ACP should be initiated before individuals lose their communication and decision-making capacities, enabling them to actively participate in discussions and decisions regarding future end-of-life care (7).

When individuals discuss end-of-life care preferences with their families and thereafter complete ADs, they are more likely to receive end-of-life care aligned with their preferences (5) and less likely to undergo nonbeneficial life-sustaining treatments (8). Among patients with dementia, AD completion is associated with reduced use of burdensome interventions, such as feeding tubes, mechanical ventilation, and cardiopulmonary resuscitation, as well as increased utilization of hospice and palliative care focused on symptom relief and comfort (9). Furthermore, AD completion has been shown to significantly reduce the emotional burden experienced by family members during surrogate decision-making processes (10).

Given these benefits, establishing effective institutional frameworks that support ACP and AD completion among older adults with dementia is essential to protect patient self-determination and improve the quality of end-of-life care. Accordingly, there is an urgent need to investigate the current status of AD completion and identify the associated multidimensional factors across different levels of cognitive function.

Previous research on AD completion has predominantly focused on the general older adult population or patients with terminal cancer; therefore, a significant gap remains regarding the specific needs of older adults with cognitive impairment (11). Older adults with cognitive decline exhibit distinct differences from their cognitively normal peers across various dimensions, including health status, chronic disease burden, medication patterns, healthcare utilization, attitudes toward death, and overall life satisfaction (12). Crucially, this cognitive and clinical heterogeneity extends into the realm of end-of-life decision making. The predictive factors and underlying mechanisms influencing AD completion among cognitively impaired older adults diverge significantly from those observed in their cognitively intact peers (13).

Furthermore, factors influencing AD completion are multi-layered, encompassing not only sociodemographic characteristics, but also clinical conditions, health behaviors, and psychological factors (such as suicidal ideation) (14, 15). Despite these complexities, there is a paucity of nationwide, large-scale comparative studies evaluating the distinct predictors of AD completion between cognitively impaired and cognitively normal subgroups. Notably, our group previously examined factors associated with AD completion among community-dwelling older Korean adults using data from the 2020 National Survey of Older Koreans (15); the present study extends this earlier work by employing the more recent 2023 survey wave and, in particular, by stratifying analyses according to cognitive status to examine whether the factors associated with AD completion differ between cognitively impaired and cognitively normal older adults, a distinction not addressed in our previous study.

To address this gap, this study utilized nationwide cross-sectional data from the 2023 National Survey of Older Koreans—a representative, population-based survey conducted every 3 years by the Korean Ministry of Health and Welfare. This study aimed to identify and compare the factors associated with AD completion—including demographic profiles, health-related characteristics, and attitudes toward death—between older adults with and without cognitive impairment, using multivariable logistic regression analysis. The findings may provide a foundation for developing cognitive status-tailored strategies to promote AD completion and for informing policies that support older adults’ right to a dignified death.

## 2 Materials and methods

### 2.1 Study design

This study was a secondary analysis of nationwide cross-sectional survey data, conducted to compare the factors associated with AD completion between older adults with cognitive impairment and those with normal cognition.

### 2.2 Data collection and participants

Data for this secondary analysis were drawn from the 2023 wave of the National Survey of Older Koreans, a nationally representative survey administered every 3 years by the Korean Ministry of Health and Welfare (16). Trained interviewers (*n* = 176) conducted face-to-face interviews using the Tablet-PC Assisted Personal Interview method between September 4 and November 12, 2023; all interviewers had prior experience with similar surveys and completed an additional 8-h training session before data collection. Eligible participants were community-dwelling adults aged 65 years or older residing in one of 17 Korean cities or provinces, identified through a proportional two-stage stratified sampling design in which the population was first stratified by the 17 metropolitan cities/provinces and then, within the nine non-metropolitan provinces, re-stratified by neighborhood (16). Sampling weights accounting for household- and individual-level nonresponse and poststratified to 2023 Statistics Korea population estimates (region, sex, age) were computed by the Ministry of Health and Welfare research team (16); however, as described in the Statistical Analysis section, these weights and the associated strata/clustering variables were not incorporated into the present analysis, which instead relied on conventional (non-survey-adjusted) multivariable logistic regression. Of the 10,078 individuals who responded to the 2023 survey, 9,951 were retained for this study after excluding 127 participants with missing cognitive-function test results; the research team received de-identified, coded data for secondary analysis.

### 2.3 Ethical considerations

The 2023 National Survey of Older Koreans was approved by Statistics Korea as a statutory survey (Approval No. 117071), and all procedures related to its data collection—including the survey instruments and informed consent process—were reviewed and approved by the Institutional Review Board of the Korea Institute for Health and Social Affairs (IRB No. 2023-078). During the original survey, IRB-approved consent forms were administered by trained investigators; participants were provided with sufficient information about the survey, their autonomy was fully respected, and written informed consent was voluntarily obtained from all participants prior to participation, in accordance with ethical standards. The present secondary-analysis protocol, which used only de-identified data, was separately reviewed and approved by the Institutional Review Board of G University (IRB No. 1044396-202506-HR-099-01), the corresponding author’s affiliated institution. This study was conducted in accordance with all applicable ethical guidelines and regulations.

### 2.4 Measurements

#### 2.4.1 Cognitive impairment

Cognitive function was assessed using the Korean version of the Mini-Mental State Examination for Dementia Screening (K-MMSE-DS) (17). Participants were classified as having cognitive impairment if their K-MMSE-DS score fell 1.5 standard deviations below the age- and education- adjusted normative mean, consistent with the cut-off criteria established for this screening instrument.

#### 2.4.2 Sociodemographic characteristics

Sociodemographic information—age (continuous, years), sex (male/female), spouse (living with a spouse: yes/no), living arrangement (living alone, married couple, household with children, or other), perceived health status (5-point scale, very healthy to very unhealthy), religion (yes/no), and educational attainment (years of formal education, continuous)—was obtained by self-report.

#### 2.4.3 Physical health-related characteristics

A broad set of physical health indicators was collected, comprising the number of diagnosed chronic diseases (continuous), number of prescribed medications used for more than 3 months (continuous), number of medical visits in the past month (continuous), alcohol consumption (yes/no, based on past-year drinking frequency), smoking status (current, former, never), nutritional status (continuous score, detailed below), body mass index (BMI, kg/m^2^, continuous), need for assistance with daily activities (a single self-reported yes/no item, distinct from the ADL/IADL classifications below), activities of daily living (ADL) and instrumental activities of daily living (IADL) (each dichotomized as fully independent vs. not fully independent, defined below), exercise frequency (days/week, continuous), history of falls in the past year (yes/no), sleep quality (5-point scale, very satisfied to not at all satisfied), registered disability status (yes/no), and functional limitation (yes/no).

Nutritional status was evaluated with the Nutritional Screening Initiative checklist (18), a 10-item, differentially weighted instrument yielding scores from 0 to 21. Following the instrument’s established cut-offs, participants were grouped into good (0–2), moderate-risk (3–5), or high-risk (≥6) nutritional categories; the continuous score was entered into the regression models.

Participants were classified as fully independent in ADL, if they reported being “fully independent” on all seven ADL items, and as fully independent in IADL if they reported being “fully independent” on all 10 IADL items; participants who did not meet this criterion on any item were classified as not fully independent.

#### 2.4.4 Emotional characteristics

Three self-reported indicators captured participants’ emotional status: depression (continuous score), satisfaction with quality of life (5-point scale, very satisfied to very dissatisfied), and past-year suicidal ideation (yes/no). Depressive symptoms specifically were measured with the 15-item Korean version of the Short Form Geriatric Depression Scale (SGDS-K) (19); items are scored dichotomously (0 or 1) and summed (range 0–15), with higher totals denoting more severe depressive symptoms. The continuous score was entered into the regression models.

#### 2.4.5 Primary outcome and attitude toward death

All items in this section were drawn from the 2023 National Survey of Older Koreans conducted by the Korean Ministry of Health and Welfare (16). AD completion was the primary outcome variable, assessed using the item, “Have you completed an advance directive in preparation for death at the end of life?” with responses coded as 1 = yes and 0 = no for the regression analyses.

Three additional indicators captured participants’ attitudes toward death: agreement with medical care for life prolongation (5-point scale, strongly agree to strongly disagree), prior participation in death preparation education (yes/no), and awareness of the significance of dying well.

The latter construct was derived from five survey items asking respondents to rate the perceived importance of specific end-of-life circumstances—namely, being able to prepare for one’s own dying process, dying free of pain, dying surrounded by family or close friends, not becoming a burden to family or close friends, and dying at home rather than in a hospital or care facility—each scored on a 5-point scale (1 = very important, 5 = not at all important) and summed into a composite ranging from 5 to 25. Because a lower rating on each item denoted greater perceived importance, a lower composite score corresponded to a higher overall level of awareness regarding the significance of dying well. This construct reflects general attitudes toward end-of-life priorities and is conceptually distinct from participants’ specific knowledge of ADs or the AD completion process, which was not separately assessed in the survey.

### 2.5 Statistical analysis

All statistical analyses were performed in IBM SPSS Statistics for Windows, version 30.0 (IBM Corp., Armonk, NY, USA); statistical significance was set at a two-tailed α of 0.05. Responses recorded as “not applicable” or “no response” were treated as missing data and handled via listwise deletion. Group comparisons between the cognitive impairment and normal cognition groups were conducted with independent-samples t-tests (continuous variables) and chi-square tests (categorical variables).

To identify factors associated with AD completion (coded as 1 = completed, 0 = not completed), multivariable binary logistic regression analyses were performed separately for the cognitive impairment group and the normal cognition group. Independent variables that showed statistically significant associations with AD completion in the bivariate analyses were entered simultaneously into each model using the forced-entry method; reference categories for categorical predictors are specified in the corresponding regression tables. Prior to fitting the regression models, multicollinearity among the independent variables was evaluated using variance inflation factors (VIF), with no evidence of severe multicollinearity detected (VIF < 10). The assumption of linearity of the logit for continuous variables was also verified. Model fit was evaluated using the Hosmer–Lemeshow goodness-of-fit test and Nagelkerke *R*^2^. Because separate regression models were fitted for each cognitive group rather than a single pooled model with interaction terms, these analyses identify within-group associations; this methodological boundary is further discussed in the Limitations section. Additionally, as noted in Section 2.2, sampling weights, stratification, and clustering from the complex survey design were not incorporated into these analyses. Because our analytic goal was to estimate within-group associations between covariates and AD completion rather than to generate population-level prevalence estimates, a conventional (non-survey-adjusted) multivariable logistic regression was considered statistically appropriate: established survey-methodology literature indicates that sampling weights are essential for unbiased descriptive or prevalence estimation, but are not necessarily required—and can reduce estimation efficiency—when the analytic objective is to estimate associations within a defined subpopulation under a correctly specified model (20–23).

## 3 Results

### 3.1 Differences in participant characteristics by cognitive impairment status

The cognitive impairment group (*n* = 2,448) and the normal cognition group (*n* = 7,503) differed significantly across sociodemographic, health-related, emotional, and attitudinal domains (Table 1). The cognitive impairment group was significantly older (74.67 ± 6.96 vs. 73.81 ± 6.67 years, *p* < 0.001), had a higher proportion of female participants (66.8% vs. 59.9%, *p* < 0.001), a higher rate of living alone (38.8% vs. 33.0%, *p* < 0.001), and lower educational attainment (7.75 ± 4.40 vs. 8.40 ± 4.17 years, *p* < 0.001) than the normal cognition group. Regarding physical health, the cognitive impairment group had more chronic conditions (2.40 ± 1.64 vs. 2.12 ± 1.58, *p* < 0.001), greater prescription medication use (2.15 ± 1.52 vs. 1.99 ± 1.61, *p* < 0.001), and more frequent healthcare visits (*p* < 0.001). The normal cognition group had higher rates of alcohol consumption (34.9% vs. 30.0%, *p* < 0.001) and current smoking (8.4% vs. 7.4%, *p* < 0.001). The cognitive impairment group was more often at moderate-to-high nutritional risk (40.3% vs. 32.1%, *p* < 0.001), had a slightly lower BMI (23.49 ± 2.72 vs. 23.69 ± 2.60, *p* < 0.001), and showed lower independence in ADL (87.8% vs. 94.3%, *p* < 0.001) and IADL (75.5% vs. 86.0%, *p* < 0.001). Conversely, the cognitive impairment group had a higher rate of needing assistance with daily activities (13.2% vs. 6.1%, *p* < 0.001) and functional limitation (24.5% vs. 14.0%, *p* < 0.001). Compared with the normal cognition group, the cognitive impairment group had higher depression scores (3.98 ± 3.50 vs. 2.80 ± 3.09, *p* < 0.001), a higher rate of suicidal ideation (1.8% vs. 0.9%, *p* = 0.001), and a higher rate of AD completion (12.5% vs. 10.7%, *p* = 0.017). The two groups also differed significantly on three of the five items assessing awareness of dying well—preparing for one’s own dying process, dying in the presence of family or close friends, and dying at home rather than in a hospital or care facility (*p* = 0.011 to *p* < 0.001)—whereas no significant differences were found for dying without pain (*p* = 0.769) or not burdening family or close friends (*p* = 0.206); item-level details are presented in Table 1.

**Table 1:** Differences in participant characteristics by cognitive impairment status (*n* = 9,951).

Categories	Variable		Cognitive impairment group (*n* = 2,448)	Normal cognition group (*n* = 7,503)	*t*/*χ*^2^	*p*
General characteristics	Age		74.67 ± 6.96	73.81 ± 6.67	5.362	<0.001
	Sex	Male	813 (33.2)	3,011 (40.1)	37.354	<0.001
	Spouse	Yes	1,321 (54.0)	4,562 (60.8)	37.73	<0.001
	Living arrangements	Living alone	950 (38.8)	2,473 (33.0)	37.658	<0.001
		Married couple	1,229 (50.2)	4,190 (55.8)		
		Household with children	243 (9.9)	697 (9.3)		
		Others	26 (1.1)	143 (1.9)		
	Perceived health status	Very healthy	42 (1.7)	198 (2.6)	220.66	<0.001
		Healthy	702 (28.7)	3,171 (42.3)		
		Moderate	885 (36.2)	2,560 (34.1)		
		Unhealthy	706 (28.8)	1,401 (18.7)		
		Very unhealthy	113 (4.6)	173 (2.3)		
	Religion	Yes	980 (40.0)	3,091 (41.2)	1.035	0.16
	Education level		7.75 ± 4.40	8.40 ± 4.17	−6.361	<0.001
Physical health-related characteristics	Number of chronic diseases		2.40 ± 1.64	2.12 ± 1.57	7.64	<0.001
	Number of prescription medications		2.15 ± 1.52	1.99 ± 1.61	4.465	<0.001
	Use of medical institutions	Fx during 1 month	1.72 ± 2.33	1.50 ± 2.18	4.127	<0.001
	Drinking alcohol	Yes	734 (30.0)	2,616 (34.9)	19.703	<0.001
	Smoking	Current	180 (7.4)	627 (8.4)	22.488	<0.001
		Past	519 (21.2)	1901 (25.3)		
		Never	1749 (71.4)	4,975 (66.3)		
	Nutritional status		2.68 ± 2.76	2.07 ± 2.39	9.748	<0.001
		Good	1,462 (59.7)	5,095 (67.9)	63.875	<0.001
		Moderate risk	697 (28.5)	1815 (24.2)		
		High risk	289 (11.8)	593 (7.9)		
	BMI		23.50 ± 2.68	23.69 ± 2.60	−3.129	0.002
	Need for assistance in daily activities	Yes	323 (13.2)	461 (6.1)	66.954	<0.001
	ADL	Fully independent	2,149 (87.8)	7,079 (94.3)	117.997	<0.001
	IADL	Fully independent	1848 (75.5)	6,455 (86.0)	148.447	<0.001
	Exercise	Days per week	2.15 ± 2.61	2.70 ± 2.71	−9.018	<0.001
	Fall experience	Yes	193 (7.9)	419 (5.6)	16.910	<0.001
	Quality of sleep	Very satisfied	80 (3.3)	239 (3.2)	103.94	<0.001
		Satisfied	932 (38.1)	3,675 (49.0)		
		Somewhat	901 (36.8)	2,438 (32.5)		
		dissatisfied	495 (20.2)	1,065 (14.2)		
		Not at all	40 (1.6)	86 (1.1)		
	Disability status	Yes	133 (5.4)	271 (3.6)	15.716	<0.001
	Functional limitation status	Yes	600 (24.5)	1,048 (14.0)	148.45	<0.001
Emotional characteristics	Depression	Yes, cutoff ≥8	3.98 ± 3.50	2.80 ± 3.09	14.893	<0.001
			412 (16.8)	690 (9.2)	109.22	<0.001
	Subjective satisfaction (QoL)	Very satisfied	25 (1.0)	123 (1.6)	228.79	<0.001
		Satisfied	679 (27.7)	3,156 (42.1)		
		Neutral	1,406 (57.4)	3,704 (49.4)		
		Dissatisfied	319 (13.0)	498 (6.6)		
		Not at all	19 (0.8)	22 (0.3)		
	Suicide intention	Yes	43 (1.8)	70 (0.9)	11.152	0.001
Attitude toward death	AD completion	Yes	305 (12.5)	804 (10.7)	5.666	0.017
	Attitude toward medical care for life prolongation	Very agree	34 (1.4)	94 (1.3)	30.552	<0.001
		Agree	101 (4.1)	333 (4.4)		
		Disagree	834 (34.1)	2,882 (38.4)		
		Very disagree	1,178 (48.1)	3,516 (46.9)		
		I do not know	301 (12.3)	678 (9.0)		
	Take death preparation education	Yes	89 (3.6)	208 (2.8)	4.752	0.029
	Awareness of dying well	Composite	9.83 ± 2.93	9.78 ± 2.90	0.779	0.436
	(1) Prepare for dying by oneself	Very important	860 (35.1)	2,490 (33.2)	13.005	0.011
		Important	1,245 (50.9)	3,895 (51.9)		
		Neutral	273 (11.2)	892 (11.9)		
		Not important	56 (2.3)	210 (2.8)		
		Not at all	14 (0.6)	16 (0.2)		
	(2) Dying without pain	Very important	1,252 (51.1)	3,740 (49.8)	1.817	0.769
		Important	846 (34.6)	2,690 (35.9)		
		neutral	283 (11.6)	881 (11.7)		
		Not important	61 (2.5)	173 (2.3)		
		Not at all	6 (0.2)	19 (0.3)		
	(3) Dying in the presence of family or close friends	Very important	658 (26.9)	2,225 (29.7)	15.266	0.004
		Important	1,194 (48.8)	3,492 (46.5)		
		Neutral	476 (19.4)	1,408 (18.8)		
		Not important	105 (4.3)	359 (4.8)		
		Not at all	15 (0.6)	19 (0.3)		
	(4) Not burdening family or close friends	Very important	1,017 (41.5)	3,236 (43.1)	5.913	0.206
		Important	1,058 (43.2)	3,094 (41.2)		
		Neutral	301 (12.3)	990 (13.2)		
		Not important	66 (2.7)	170 (2.3)		
		Not at all	6 (0.2)	13 (0.2)		
	(5) Dying at home rather than in a hospital or care facility	Very important	270 (11.0)	955 (12.7)	19.608	<0.001
		Important	962 (39.3)	3,074 (41.0)		
		Neutral	866 (35.4)	2,370 (31.6)		
		Not important	296 (12.1)	869 (11.6)		
		Not at all	54 (2.2)	235 (3.1)		

fx, frequency; BMI, body mass index; ADL, activities of daily living; IADL, instrumental activities of daily living; ADs, advance directives; QoL, quality of life.

### 3.2 Differences in participant characteristics by AD completion among older adults with cognitive impairments

Within the cognitive impairment group (*n* = 2,448), participants who completed an AD differed significantly from those who had not across several characteristics (Table 2). Participants who completed an AD were significantly younger (*p* < 0.001), more likely to have a spouse (*p* = 0.032), more likely to have a religious affiliation (*p* < 0.001), and had higher educational attainment (*p* < 0.001). Those who completed an AD also had more chronic conditions and greater prescription medication use (*p* < 0.001), and more frequent healthcare visits (*p* = 0.037), as well as higher rates of past or current smoking (*p* < 0.001) and more frequent exercise (*p* < 0.001). They were more likely to have depression (*p* < 0.001) and suicidal ideation (*p* = 0.002), had higher participation in death preparation education (*p* < 0.001), and had lower scores on the dying-well awareness scale (*p* < 0.001).

**Table 2:** Differences in participant characteristics by AD completion in older adults with cognitive impairment (*n* = 2,448).

Categories	Variable		Older adults who completed ADs (*n* = 305)	Older adults who did not completed ADs (*n* = 2,143)	*t*/*χ*^2^	*p*
General characteristics	Age		72.91 ± 5.99	74.92 ± 7.05	−5.367	<0.001
	Sex	Male	113 (37.0)	700 (32.7)	2.314	0.135
	Spouse	Yes	182 (59.7)	1,139 (53.1)	4.572	0.032
	Living arrangements	Living alone	112 (36.7)	838 (39.1)	19.081	<0.001
		Married couple	179 (58.7)	1,050 (49.0)		
		Household with children	14 (4.6)	229 (10.7)		
		Others	0(0.0)	26(1.2)		
	Perceived health status	Very healthy	7 (2.3)	35 (1.6)	1.759	0.78
		Healthy	83 (27.2)	619 (28.9)		
		Moderate	106 (34.8)	779 (36.4)		
		Somewhat unhealthy	93 (30.5)	613 (28.6)		
		Unhealthy	16 (5.2)	97 (4.5)		
	Religion (Yes)	Yes	168 (55.1)	812 (37.9)	32.869	<0.001
	Education level		9.47 ± 4.35	7.51 ± 4.35	7.378	<0.001
Physical health-related characteristics	Number of chronic diseases		2.79 ± 1.41	2.35 ± 1.67	5.025	<0.001
	Number of prescription medications		2.57 ± 1.41	2.10 ± 1.53	5.084	<0.001
	Use of medical institutions	Fx during 1 month	1.98 ± 2.08	1.68 ± 2.36	2.089	0.037
	Drinking alcohol	Yes	103 (33.8)	631 (29.4)	27.418	<0.001
	Smoking	Current	21 (6.9)	159 (7.4)	19.392	<0.001
		Past	94 (30.8)	425 (19.8)		
		Never	190 (62.3)	1,559 (72.7)		
	Nutritional status		3.62 ± 2.73	2.54 ± 2.74	6.42	<0.001
	BMI		23.54 ± 2.67	23.48 ± 2.73	0.37	0.356
	Need for assistance in daily activities	Yes	32 (10.5)	291 (13.6)	2.477	0.29
	Exercise	Days per week	3.11 ± 2.69	2.01 ± 2.57	6.708	<0.001
	Fall experience	Yes	33 (10.8)	160 (7.5)	4.135	0.042
	ADL	Fully independent	272 (89.2)	1877 (87.8)	0.632	0.427
	IADL	Fully independent	240 (78.7)	1,608 (75.0)	1.926	0.165
	Disability status	Yes	18 (5.9)	115 (5.4)	0.149	0.7
	Functional limitation status	Yes	65 (21.3)	535 (25.0)	1.926	0.165
Emotional characteristics	Depression	Yes	76 (24.9)	336 (15.7)	16.282	<0.001
	Suicide intention	Yes	12 (3.9)	31 (1.4)	9.576	0.002
Attitude toward death	Take death preparation education	Yes	30 (9.8)	59 (2.8)	38.233	<0.001
	Awareness of dying well	Composite	8.94 ± 2.48	9.96 ± 2.97	−6.533	<0.001

fx, frequency; BMI, body mass index; ADL, activities of daily living; IADL, instrumental activities of daily living.

### 3.3 Differences in participant characteristics by AD completion among older adults without cognitive impairments

In the normal cognition group (*n* = 7,503), participants who completed an AD also differed significantly from those who had not (Table 3). They were significantly older (*p* = 0.005), more likely to live alone (*p* = 0.032), more likely to have a religious affiliation (*p* < 0.001), and had more chronic diseases and prescription medications (*p* < 0.001) than those who had not. Rates of alcohol consumption did not differ significantly between the two groups (*p* = 0.465), although those who completed an AD had lower rates of smoking (*p* = 0.002). As in the cognitive impairment group, those who completed an AD reported poorer nutritional status (*p* < 0.001), lower BMI (*p* = 0.026), more frequent exercise (*p* < 0.001), more falls (*p* < 0.001), and higher depression (*p* = 0.007). They also had higher participation in death preparation education (*p* < 0.001) and lower dying-well awareness scores (*p* < 0.001).

**Table 3:** Differences in participant characteristics by AD completion in older adults without cognitive impairment (*n* = 7,503).

Categories	Variable		Older adults who completed ADs (*n* = 804)	Older adults who did not completed ADs (*n* = 6,699)	*t*/*χ*^2^	*p*
General characteristics	Age		74.44 ± 6.41	73.74 ± 6.70	2.838	0.005
	Sex	Male	313 (38.9)	2,698 (40.3)	0.54	0.47
	Spouse	Yes	496 (61.7)	4,066 (60.7)	0.299	0.593
	Living arrangements	Living alone	271 (33.7)	2,202 (32.9)	8.839	0.032
		Married couple	468 (58.2)	3,722 (55.6)		
		Household with children	54 (6.7)	643 (9.6)		
		Others	11 (1.4)	132 (2.0)		
	Perceived health status	Very healthy	23 (2.9)	175 (2.6)	8.042	0.09
		Healthy	331 (41.2)	2,840 (42.4)		
		Moderate	266 (33.1)	2,294 (34.2)		
		Somewhat unhealthy	173 (21.5)	1,228 (18.3)		
		Unhealthy	11 (1.4)	162 (2.4)		
	Religion	Yes	408 (50.7)	2,683 (40.1)	33.898	<0.001
	Education level	Years	8.62 ± 4.31	8.37 ± 4.16	1.562	0.119
Physical health-related characteristics	Number of chronic diseases		2.40 ± 1.58	2.09 ± 1.57	5.393	<0.001
	Number of prescription medications		2.18 ± 1.52	1.97 ± 1.62	3.51	<0.001
	Use of medical institutions	Fx during 1 month	1.62 ± 2.26	1.49 ± 2.17	1.676	0.094
	Drinking alcohol	Yes	271 (33.7)	2,345 (35.0)	0.533	0.465
	Smoking	Current	42 (5.2)	585 (8.7)	12.695	0.002
		Past	223 (27.7)	1,678 (25.0)		
		Never	539 (67.0)	4,436 (66.2)		
	Nutritional status		2.40 ± 2.51	2.03 ± 2.37	3.967	<0.001
	BMI		23.50 ± 2.59	23.72 ± 2.60	−2.234	0.026
	Need for assistance in ADL	Yes	54 (6.7)	407 (6.1)	0.511	0.475
	Exercise	Days per week	3.45 ± 2.75	2.61 ± 2.69	8.397	<0.001
	Fall experience	Yes	74 (9.2)	345 (5.2)	22.375	<0.001
	ADL	Fully independent	745 (92.7)	6,334 (94.6)	4.808	0.02
	IADL	Fully independent	679 (84.5)	5,776 (86.2)	1.87	0.178
	Disability status	Yes	39 (4.9)	232 (3.5)	3.97	0.057
	Functional limitation status	Yes	125 (15.5)	923 (13.8)	1.87	0.178
Emotional characteristics	Depression	Yes	95 (11.8)	595 (8.9)	7.4	0.007
	Suicide intention	Yes	8 (1.0)	62 (0.9)	0.038	0.845
Attitude toward death	Takes death preparation education	Yes	85 (10.6)	123 (1.8)	203.26	<0.001
	Awareness of dying well		9.10 ± 2.54	9.86 ± 2.93	7.86	<0.001

fx, frequency; BMI, body mass index; ADL, activities of daily living; IADL, instrumental activities of daily living.

### 3.4 Factors associated with AD completion in those with cognitive impairments

Multivariable logistic regression analysis was conducted to identify factors associated with AD completion in the cognitive impairment group (Table 4). AD completion was significantly associated with younger age (adjusted odds ratio [aOR] = 0.965, 95% CI = 0.941–0.989), female sex (aOR for male sex = 0.399, 95% CI = 0.229–0.695), religious affiliation (aOR = 1.748, 95% CI = 1.336–2.286), higher education (aOR = 1.099, 95% CI = 1.057–1.142), greater prescription medication use (aOR = 1.202, 95% CI = 1.053–1.371), never having smoked relative to current smoking (aOR = 0.457, 95% CI = 0.231–0.904), poorer nutritional status (aOR = 1.146, 95% CI = 1.089–1.206), higher exercise frequency (aOR = 1.162, 95% CI = 1.106–1.220), higher ADL independence (aOR = 1.715, 95% CI = 1.082–2.719), higher depressive symptom scores (aOR = 1.057, 95% CI = 1.010–1.105), participation in death preparation education (aOR = 3.300, 95% CI = 1.991–5.470), and higher awareness of dying well, reflected by a lower total score (aOR = 0.866, 95% CI = 0.820–0.915).

**Table 4:** Factors associated with AD completion in older individuals with cognitive impairments (*n* = 2,448).

Independent variable		B	SE	*p*	Adjusted OR	95% CI	
						Lower	Upper
Age		−0.039	0.012	0.002	0.962	0.939	0.985
Sex	Male	−0.919	0.283	0.001	0.399	0.229	0.695
Spouse	Yes	0.205	0.144	0.155	1.228	0.926	1.628
Religion	Yes	0.558	0.137	<0.001	1.748	1.336	2.286
Education level		0.094	0.020	<0.001	1.099	1.057	1.142
Number of chronic diseases		−0.022	0.067	0.746	0.978	0.858	1.116
Number of prescribed medications		0.184	0.067	0.006	1.202	1.053	1.371
Number of medical visits in the past month		0.032	0.028	0.262	1.032	0.976	1.092
Alcohol use	Yes	0.098	0.159	0.539	1.103	0.807	1.506
Smoking (current)	Past	0.463	0.281	0.100	1.589	0.915	2.758
	Never	−0.782	0.348	0.024	0.457	0.231	0.904
Nutritional status		0.136	0.026	<0.001	1.146	1.089	1.206
Exercise (fx)	During 1 week	0.150	0.025	<0.001	1.162	1.106	1.220
Fully independent ADL	Yes	0.540	0.235	0.022	1.715	1.082	2.719
Depression	Yes	0.055	0.023	0.016	1.057	1.010	1.105
Death preparation education	Yes	1.194	0.258	<0.001	3.300	1.991	5.470
Suicide intention	Yes	0.541	0.416	0.193	1.718	0.760	3.882
Awareness of dying well		−0.143	0.028	<0.001	0.866	0.820	0.915
Nagelkerke *R*^2^ = 0.211							

SE, standard error; OR, odds ratio; CI, confidence interval; fx, frequency; ADL, activities of daily living. Awareness of dying well was scored such that lower total scores indicate greater perceived importance of dying well; thus, an aOR < 1 indicates that higher awareness of dying well was associated with a greater likelihood of AD completion.

Hosmer & Lemeshow test 15.204 (*p* = 0.055).

### 3.5 Factors associated with AD completion in those without cognitive impairments

Multivariable logistic regression analysis was conducted to identify factors associated with AD completion in the normal cognition group (Table 5). Unlike in the cognitive impairment group, sex was not significantly associated with AD completion in this group (aOR = 1.010, 95% CI = 0.847–1.204, *p* = 0.913). AD completion was significantly associated with religious affiliation (aOR = 1.448, 95% CI = 1.242–1.689), a higher number of chronic diseases (aOR = 1.151, 95% CI = 1.063–1.246), and poorer nutritional status (aOR = 1.050, 95% CI = 1.014–1.087). Higher exercise frequency (aOR = 1.118, 95% CI = 1.087–1.150) and a history of falls within the past year (aOR = 1.647, 95% CI = 1.245–2.178) were also significant physical health–related correlates, whereas BMI showed only a borderline, non-significant association (aOR = 0.971, 95% CI = 0.942–1.001, *p* = 0.054). Participation in death preparation education (aOR = 6.198, 95% CI = 4.599–8.354) and higher awareness of dying well, reflected by a lower total score (aOR = 0.904, 95% CI = 0.878–0.931) were also significantly associated with AD completion in this group.

**Table 5:** Factors associated with AD completion in older individuals without cognitive impairments (*n* = 7,503).

Independent variable		B	SE	*p*	Adjusted OR	95% CI	
						Lower	Upper
Age		0.012	0.006	0.051	1.012	1.000	1.025
Sex	Male	0.010	0.090	0.913	1.010	0.847	1.204
Living alone	Yes	−0.068	0.088	0.439	0.934	0.787	1.110
Religion	Yes	0.370	0.078	<0.001	1.448	1.242	1.689
Number of chronic diseases		0.141	0.040	<0.001	1.151	1.063	1.246
Number of prescribed medications		−0.070	0.040	0.081	0.932	0.861	1.009
Alcohol use	Yes	0.072	0.090	0.428	1.074	0.900	1.283
Nutritional status		0.049	0.018	0.006	1.050	1.014	1.087
BMI		−0.030	0.015	0.054	0.971	0.942	1.001
Exercise (fx)	During 1 week	0.112	0.014	<0.001	1.118	1.087	1.150
Fall experience during the past 1 year	Yes	0.499	0.143	<0.001	1.647	1.245	2.178
Death preparation education (yes)	Yes	1.824	0.152	<0.001	6.198	4.599	8.354
Independent ADL	Yes	0.086	0.167	0.607	1.090	0.785	1.512
Awareness of dying well		−0.101	0.015	<0.001	0.904	0.878	0.931
Nagelkerke *R*^2^ = 0.087							

SE, standard error; OR, odds ratio; CI, confidence interval; BMI, body mass index; fx, frequency; ADL, activities of daily living. Awareness of dying well was scored such that lower total scores indicate greater perceived importance of dying well; thus, an aOR < 1 indicates that higher awareness of dying well was associated with a greater likelihood of AD completion.

Hosmer & Lemeshow test 7.091 (*p* = 0.527).

## 4 Discussion

In this study, cognitive impairment was defined as a score on the K-MMSE-DS that was 1.5 standard deviations or more below the mean for the participant’s respective age and education group (16). As a result, 24.6% of the entire sample was determined to have cognitive impairment. With medical advancements improving the possibility of having a prolonged life, people increasingly seek the right to self-determination regarding both life and death (24). When caring for end-of-life patients, ACP helps clinicians and families honor and implement the patient’s preferences (24). As South Korea has transitioned into a super-aged society due to the rapidly increasing older adult population, AD completion is becoming increasingly important (25). However, according to this study, only 12.5% of older adults in the cognitively impaired group and 10.7% in the cognitively normal group had completed an AD.

The results of this study demonstrate that end-of-life education is strongly associated with higher odds of AD completion, regardless of cognitive function levels. Notably, in the normal cognition group, the aOR associated with death preparation education was approximately twice as high as that observed in the cognitive impairment group. Furthermore, a staggering 90.3% of individuals have never independently explored life-sustaining treatment systems or AD completion (21). In 2025, the registration rate for AD completion among South Korean adults aged ≥19 years was 6.8% (26). Although 82.2% of participants in the cognitive impairment group and 85.3% in the normal cognition group in this study did not agree with medical care for life prolongation, the overall rate of AD completion remained low. This discrepancy suggests that while negative attitudes toward non-beneficial life extension are widespread, personal willingness does not automatically translate into formal documentation. This low completion rate may reflect the fact that factors associated with AD completion are fundamentally multi-layered—encompassing not only sociodemographic characteristics, but also clinical conditions, health behaviors, psychological states, and structural accessibility (14, 15). Consequently, addressing this low completion rate requires going beyond generalized educational programs to consider these complex, multi-layered determinants, which may operate differently depending on cognitive status. Furthermore, religious affiliation, poorer nutritional status, higher exercise frequency, and higher awareness of the significance of dying well were identified as significantly associated with AD completion in both the groups. However, in a previous study, 75.1% of community-dwelling older adults have never been introduced to the formal concept of “dying well” (27). While greater perceived importance of dying well was associated with AD completion, the exact underlying mechanism remains unclear. One possible explanation is that prioritizing end-of-life values may foster general receptivity toward advance care planning; however, systematic exposure to these concepts remains low, highlighting an area that warrants further empirical investigation.

Several additional factors were significantly associated with AD completion within the cognitive impairment group, including a complex interplay of sociodemographic factors (such as age and educational attainment), number of prescription medications used, independence in ADL, and depressive symptoms. A particularly notable group-specific finding was sex: female sex was significantly associated with a higher likelihood of AD completion in the cognitive impairment group (aOR for male sex = 0.399, 95% CI = 0.229–0.695), whereas sex showed no significant association with AD completion in the normal cognition group (aOR = 1.010, 95% CI = 0.847–1.204). Prior research on sex differences in AD completion has yielded mixed and context-dependent findings: a study of community-dwelling Korean older adults reported higher AD completion among men than women, a pattern the authors themselves noted was inconsistent with earlier Korean studies reporting higher completion among women (15). Similarly, a dyadic study of older American couples found that the association between cognitive function and AD completion differed according to spousal sex, suggesting that gender and cognitive status may jointly shape advance care planning behavior (28). Although the present study did not formally test for a statistical interaction between sex and cognitive status, our finding that sex was significantly associated with AD completion only in the cognitively impaired group—but not in the cognitively normal group—adds to this body of evidence suggesting that the role of sex in AD completion may not be uniform across populations and may depend on cognitive status, warranting further investigation through studies formally designed to test sex-by-cognition interactions. Regarding depression, the association with AD completion in the cognitive impairment group was statistically significant but modest in magnitude (aOR = 1.057, 95% CI = 1.010–1.105). This finding is broadly consistent with prior literature indicating that depression is more prevalent among individuals with cognitive impairment, reaching up to 32% (29), and is recognized as a factor associated with perceptions of mortality and death anxiety in older adults (29, 30). However, given the cross-sectional design and modest effect size observed here, this association should be interpreted as preliminary, and the underlying mechanism cannot be established from the present data.

In the cognitively normal group, a higher number of chronic diseases and a history of falls were significantly associated with increased odds of AD completion. A history of falls is associated with fear of falling among older adults (31), which in turn negatively affects quality of life (32). For cognitively intact older adults, these physical health factors may highlight bodily decline and prompt earlier engagement with advance directives; however, this interpretation is a hypothesis that was not directly tested in the present cross-sectional study and warrants further investigation, for example through longitudinal designs. In contrast, these physical factors showed no significant association with AD completion among older adults with cognitive impairment, indicating that key determinants of advance care planning may vary according to cognitive status.

Strategies to encourage AD completion may need to be tailored to individuals’ level of cognitive function. Specifically, advance directive education and communication for older adults with cognitive impairment may benefit from sensitively accounting for sociodemographic factors, functional status, depressive symptoms, and—based on the present findings—sex. Conversely, educational approaches for cognitively intact individuals might leverage current health status and intact functional capacities. Overall, these findings highlight the potential value of tailored death education programs and suggest that communication strategies to encourage AD completion warrant further refinement and empirical testing.

### 4.1 Strengths and limitations

This study has notable strengths. It used data from a large, nationally representative sample of community-dwelling older Koreans, which allowed factor associated with AD completion to be examined separately among individuals with and without cognitive impairment. To our knowledge, this is among the first studies to explore these factors by cognitive status using a nationwide dataset, and the findings may inform future efforts to tailor advance care planning approaches to individuals’ cognitive status.

However, several limitations warrant consideration. First, because of the cross-sectional nature of the dataset, causal relationships between the identified factors and AD completion could not be established. Longitudinal studies are required to clarify the temporal relationships and potential causal pathways underlying AD completion, particularly in relation to cognitive status. Second, as a secondary data analysis, the findings were restricted to variables available in the original dataset. Other potentially relevant factors—such as cognitive and psychosocial processes and individuals’ knowledge or awareness of ADs—were not captured. Given that awareness of ADs is likely to influence completion, the lack of this measure may have resulted in residual confounding. Future studies incorporating theory-driven variables, including AD-specific knowledge, are warranted. Third, only community-dwelling older adults who were able to provide self-reported data were included. Participants classified as cognitively impaired were therefore likely to have relatively mild impairment, and the findings may not be generalizable to older adults with more severe cognitive impairment or to those in institutional or hospital settings. Future studies should include more diverse populations across different care settings. Fourth, cognitive impairment was determined using a cut-off score on a cognitive screening instrument (K-MMSE-DS) rather than a comprehensive clinical diagnostic assessment. Participants classified as cognitively impaired may not have had a clinically confirmed diagnosis of dementia or mild cognitive impairment, and such misclassification may have attenuated differences between groups. More robust diagnostic assessments should be used in future research. Fifth, unweighted multivariable logistic regression was used rather than complex survey design adjustments. While suitable for assessing within-group covariate associations, omitting sampling weights may affect standard error precision and limit the generalizability of population-level descriptive prevalence estimates. Finally, because factors associated with AD completion were examined using separate regression models for each cognitive group, these within-group findings do not statistically confirm between-group differences. Future research should incorporate formal interaction analyses to directly test for moderation by cognitive status.

## 5 Conclusion

This study demonstrated that different patterns of factors associated with AD completion were observed between cognitively impaired and cognitively normal older adults, although several factors were common to both the groups. Religious affiliation, nutritional status, exercise frequency, participation in death preparation education, and higher awareness of dying well were significantly associated with AD completion in both cohorts.

However, several factors were uniquely associated with AD completion within each cognitive group. In the cognitive impairment group, younger age, female sex, higher educational attainment, greater prescription medication use, never having smoked, higher ADL independence, and higher depressive symptom scores were significantly associated with AD completion. In the normal cognition group, a greater number of chronic diseases and a recent history of falls were uniquely associated with AD completion; notably, sex was not significantly associated with AD completion in this group, in contrast to the cognitive impairment group.

These findings suggest that the factors associated with AD completion may differ according to cognitive status, indicating that strategies to promote AD completion may need to be tailored accordingly. For older adults with cognitive impairment, approaches that address functional independence, psychosocial factors such as depressive symptoms may warrant particular attention. In addition, men with cognitive impairment may warrant closer attention in advance care planning discussions, although the effectiveness of sex-tailored approaches requires further investigation. For cognitively normal older adults, the association between falls and chronic disease burden with AD completion suggests that routine health encounters—such as those following a fall or a new diagnosis—may represent opportunities to introduce AD-related discussions, although this hypothesis was not directly tested in this study and warrants further investigation.

## Data Availability

The original contributions presented in the study are included in the article/supplementary material, further inquiries can be directed to the corresponding author.
